# Executive Functioning and Clinical Variables in Patients with Obsessive-Compulsive Disorder

**DOI:** 10.3390/brainsci11020267

**Published:** 2021-02-20

**Authors:** Inmaculada Concepción Martínez-Esparza, Pablo J. Olivares-Olivares, Ángel Rosa-Alcázar, Ana I. Rosa-Alcázar, Eric A. Storch

**Affiliations:** 1 Department of Personality, Assessment & Psychological Treatment, University of Murcia, 30100 Murcia, Spain; inmaculada.me@gmail.com (I.C.M.-E.); pjoo1@um.es (P.J.O.-O.); 2 Department of Psychology, Catholic University of Murcia, 30107 Murcia, Spain; aralcazar@ucam.edu; 3 Department of Psychiatry and Behavioral Sciences, Baylor College of Medicine, Houston, TX 77030, USA; eric.storch@bcm.edu

**Keywords:** obsessive–compulsive disorder, executive function, cognitive flexibility, inhibition, working memory

## Abstract

Background: Cognitive flexibility, response inhibition, and working memory are considered the main mechanisms responsible for executive control. This study examined differences in cognitive flexibility, inhibition, and working memory in patients with obsessive–compulsive disorder (OCD) relative to a control group. Method: A total of 62 obsessive-compulsive participants (OCD = 32; healthy control = 32) aged between 17 and 56 years old (M = 33.16, SD = 9.23) were administered the computerized Wisconsin Card Sorting Test, Stroop Color–Word Test, Go/No-Go Task, Digit Test, and Corsi Block Test. Clinician-rated and self-reported obsessive–compulsive symptom severity, and anxiety, depression, and obsessive beliefs were evaluated. Results: The control group performed better than the OCD group in tasks involving cognitive flexibility, inhibition, and visuospatial working memory. Anxiety and obsessive beliefs influenced the participants’ performance on inhibition and working memory tasks. Similarly, comorbidity also influenced inhibition and working memory. In addition, the use of pharmacotherapy and the degree of OCD symptom severity influenced verbal working memory. Conclusions: Cognitive flexibility, inhibition, and visuospatial working memory deficits may be endophenotypes of OCD but require further examination for specificity. OCD severity, comorbidity patterns, anxiety, and obsessive beliefs may influence performance.

## 1. Introduction

Obsessive–compulsive disorder (OCD) is an impairing psychiatric condition characterized by the occurrence of thoughts, recurrent images or impulses, and/or compulsions aimed at reducing discomfort or preventing feared events [1]. Given the heterogeneous nature of OCD and its multidetermined etiology, research in neuropsychology has attempted to ascertain the degree of correspondence between neuroimaging data and neuropsychological test results, with the purpose of identifying a clinical phenotype, and to predict and improve treatment outcomes [2].

Executive function (EF), and the relationship between its different subdomains as independent entities or as part of a whole [3], has been the subject of several studies in OCD. EF consists of regulatory mechanisms of cognition, behavior, and emotions that allow the achievement of individual goals and objectives [4]. Currently, cognitive flexibility (CF), response inhibition (RI), and working memory (WM) are considered the main mechanisms responsible for executive control [5]. 

Correspondingly, CF is the ability that allows changing representation based on incoming information, and to keep representation intact when changes are irrelevant [6]. The Wisconsin Card Sorting Test (WCST) is the most frequently used test for assessing this EF. Perseverative errors are the main signs of frontal dysfunction [7]. RI refers to mental processes responsible for intentional and voluntary control, or the ability to prevent interference of non-pertinent information in the face of responses or patterns of responses underway, and to suppress previously relevant information which is not currently useful [8]. RI is not considered a unitary function, as it includes RI at the motor (or behavioral) level and the control of interference or cognitive inhibition. Some tests are the Stroop Test and Go/No-Go Task. WM is defined as maintaining or manipulating information across a short delay when that information is not available in the environment and involves both short-term storage of information and simultaneous manipulation of mental content [9]. WM can also be divided into verbal and visuospatial components [10]. 

Researchers have reported neuropsychological deficits as a primary characteristic of patients with OCD [11,12]. Saremi et al. [13] concluded that these deficits have clinical significance in understanding incidence, maintenance, and severity of OCD symptoms. CF has been lower in those with OCD when compared to healthy participants [14]. Research in RI and WM in OCD patients has yielded conflicting results. Some studies report a worse performance for errors of commission in participants with OCD in a Go/No-Go Task (motor inhibition), with no differences in errors of omission and reaction time [15,16]. Other studies have found differences in both errors of commission and reaction time [15], while Kurt et al. [17] found no differences. 

In addition, worse performance in WM tasks has been observed in OCD patients when compared to a control group [18]. Other findings have yielded contradicting results indicating there were no differences between OCD and control groups [19,20]. Differences in WM remain significant even after the controlling effects of age, gender, and education [21], obtaining worse results in participants with the highest age at onset [22].

There is limited knowledge on OCD subtypes and their relationship to neuropsychological findings. Saremi et al. [13] suggested that obsessive–compulsive (OC) washers performed significantly worse on neuropsychological measures than controls. There was no significant association between severity of OC symptoms and neuropsychological functions in the OCD washing group. Rasmussen et al. [23] concluded that there were no deficits in RI in OCD scrupulousness and contamination groups compared to healthy controls. Bragdon et al. [24] assessed the “symmetry” versus “obsessing” dimension (S/O), finding that increased S/O symptoms were associated with a worse performance in verbal WM. 

Other variables related to EF are symptom severity, duration of illness, and age of onset [16,25]. However, other studies have reported the absence of a significant association between symptom severity and performance in neuropsychological testing [26]. Some findings suggest that performance is independent of symptom severity but dependent on duration of illness [22]. 

However, cognitive models propose that dysfunctional beliefs and intrusive thoughts play a central role in maintaining mental disorders [27]. Bradbury et al. [28] stated that the importance attached to obsessive beliefs in OCD could cause deficits in CF. Additionally, Şahin et al. [29] found greater cognitive inflexibility in OCD patients with higher obsessive beliefs. Likewise, findings from Grisham and Williams [30] indicated that participants in an OCD group displayed a greater need to control their thoughts when compared to social anxiety and healthy groups, which could be related to worse results obtained in WM and RI.

Multiple meta-analyses have attempted to elucidate these discrepancies. For instance, Abramovitch et al. [31] reported that RI showed a lesser overall effect size than expected, perhaps due to the different sensitivity of tests used (Go/No-Go errors of commission and Stroop interference). Similar constraints were observed with CF and WM results, alluding to the impact that tool selection can have on research findings and important underlying limitations in primary studies. Other meta-analyses and systematic reviews [11,12] suggest that effect sizes were medium in CF, medium and medium-low in RI, while low and medium in WM. Abramovitch et al. [25] found that the worst performance of OCD patients in neuropsychological tests was linked to symptomatic severity approaching a mean effect size in CF tasks. However, they indicated that there is a need to develop a psychometrically valid measure of OCD symptom severity at the time of the test, or to assess the general psychopathological burden in parallel to OCD.

The meta-analyses previously discussed highlight the limitations in primary studies. In particular, they reported that some studies had used self-report measures rather than clinical interviews; anxiety and depression had not been controlled with validated quantitative measures. Furthermore, variables that could influence results such as OCD subtypes, comorbidity, beliefs obsession, use of medication, age and sex, duration of disorder, and age of onset were not controlled [11,12,25,31]. Thus, the inconsistencies across neuropsychological studies on patients with OCD warrant a closer examination on potential confounding factors of neuropsychological function. 

### Study Aims

The aim of the present study is to examine differences in CF, RI, and WM in patients with OCD versus healthy controls. Specifically, the study aimed to (1) examine CF, RI, and WM differences among patients with OCD and a healthy CG; (2) examine the relationship between comorbid disorders and CF, RI, and WM performance; (3) evaluate the effects of comorbidity, OCD subtypes, and medication use on EF in the OCD group; and (4) assess the relationship between EF and symptom severity and OCD duration. 

## 2. Material and Method

### 2.1. Participants

There were 62 participants between the ages of 17 and 56 years (Mean = 33.16, SD = 9.23) diagnosed with OCD [1] and a healthy CG (control group). The study sample was recruited from both clinical and community settings. Recruitment was conducted through advertising by the Applied Psychology Service of the University of Murcia and through multiple mental health centers in the regions of Murcia and Castilla-La Mancha (Spain). Once the clinical group was established, the CG was recruited to be equal in age, sex, and educational level. Primary obsessions were aggressive (31.3%), miscellaneous, superstitions (28.1%), contamination (18.8%), somatic (12.5%), symmetry or exactness (6.3%), and sexual (3.1%). Primary compulsions were checking (65.6%), cleaning/washing (15.6%), ordering/arranging (9.4%), repeating (6.3%), and miscellaneous (3.1%). The average duration of OCD was 11.5 years. The OCD participants with comorbidity was 16. Mean dose antidepressants and antipsychotics administered to the participants were sertraline, 100 mg/d; fluoxetine, 40.5 mg/d; fluvoxamine, 200 mg/d; citalopram, 20 mg/d; and haloperidol, 1 mg/d.

Inclusion criteria for participants in the clinical group included (1) a diagnosis of OCD; (2) a Yale–Brown Obsessive Compulsive Scale (Y-BOCS) [32] total score ≥16; and between 16 and 65 years of age. Exclusion criteria included comorbidity with bipolar disorder, schizophrenia spectrum disorders, psychotic disorders, personality disorders, anorexia, bulimia, substance abuse disorders, and neurocognitive disorders. Participants in the CG were excluded if (1) they presented with a current psychopathological disorder; (2) had experienced or been diagnosed at some point in their life with OCD, generalized anxiety disorder (GAD), or any of the aforementioned disorders; and (3) they had a family history of any of the previously mentioned disorders per patient report. A total of 105 participants from the community population volunteered for inclusion in the research with 55 discarded due to exclusion criteria. With the remaining 50, a match was made according to age, sex, and educational level with the OCD group. Eighteen could not be matched. Two men from the CG did not finish the study.

Sample characteristics are presented in Table 1.

### 2.2. Procedure 

The study met the ethical standards of the Declaration of Helsinki and has been approved by the Ethics Committee of the University of Murcia, Spain (code: 1296/2016). All participants provided written informed consent. 

**Figure 1:**
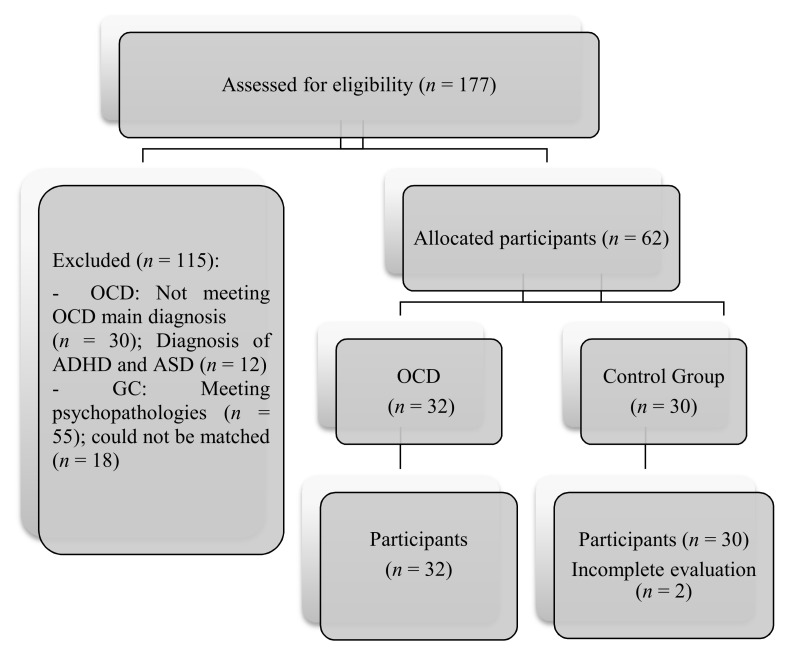
CONSORT flow diagrams of study development.

After providing written consent, participants engaged in an individual diagnostic interview based on DSM-5 criteria (SCID-I, SCID-II), conducted by three clinical psychologists. Assessment was conducted in two 60-min sessions by three experienced clinical psychologists who were trained by the fourth author for two sessions of one hour each. The test presentation order was the same for all participants. Participation in this study was voluntary and participants could discontinue at any given time. Recruitment is shown in Figure 1.

### 2.3. Measures

#### 2.3.1. Clinical Measures

The Yale–Brown Obsessive Compulsive Scale (Y-BOCS) [32] is comprised of 10 items assessing the severity of OCD. It contains two subscales, obsessions (range = 0–20) and compulsions (range = 0–20), and a total score (range = 0–40). The scale has a high internal consistency (*α* = 0.87–0.90) and good convergent validity (*r* = 0.47–0.74). Cronbach’s alpha in this study was 0.87.

Beck-II Depression Inventory (BDI) [33] is a 21-item self-report scale that assesses depression severity. Classification of scores was as follows: minimal (0 to 13), mild (14 to 19), moderate (20 to 28), and severe (>29). Cronbach’s alpha in this study was 0.91.

Beck Anxiety Inventory (BAI) [34] is a 21-item self-report scale that measures anxiety severity and level. Classification of scores was as follows: minimal (0 to 7), mild (8 to 15), moderate (16 to 25), and severe (26+). The internal consistency coefficients varied between 0.85 and 0.93. Cronbach’s alpha was 0.92.

The Obsessive Beliefs Spanish Inventory-Revised (OBSI-R) [35] is a 50-item self-report questionnaire (from 1, strongly disagree, to 7, strongly agree) with eight scales: inflated responsibility, over-importance of thoughts, thought action fusion-likelihood, thought action fusion-moral, importance of controlling one’s thoughts, overestimation of threat, intolerance of uncertainty, and perfectionism. This has been shown to have adequate psychometric properties. Cronbach’s alpha was 0.92.

#### 2.3.2. Neuropsychological Measures

The Wisconsin Card Sorting Test (WCST) [36] assesses CF or attentional change using a set of cards. The most important measures are number of categories completed, perseverative responses, total errors, perseverative errors, and non-perseverative errors. The T-score is used taking into account age and educational level. The psychometric properties of the WCST have been widely researched, oscillating Cronbach’s alpha reliability coefficients between 0.39 and 0.72.

The Stroop Color–Word Test (SCWT) [37] assesses the ability to inhibit the automatic tendency to respond verbally and, therefore, control response to conflicting stimuli (words, colors, words/colors, and interference). The test–retest reliability was (Pearson’s *r* = 0.85, *r* = 0.81, *r* = 0.69).

The Go/No-Go Task [38] evaluates motor RI. It involves two stimuli (arrows of different colors and positions), one requiring a response (Go), and one requiring no response (No/Go). This presented good convergent validity in this study, 0.87.

The Digit Span Test [39] has three parts: digit forwards, digit backwards, and increasing digit. The most important measure is the maximum number of elements (SPAN) that the individual can remember short term in reverse or increasing order. Reliability and validity indices were adequate. 

The Corsi Block Task WMS-III (Wechsler-III Memory Scale) [40] evaluates visuospatial short-term memory and consists of a forward and backward task. The most important data are the SPAN backward. It showed appropriate reliability and validity indices.

### 2.4. Data Analysis

Firstly, a Chi-square and one-factor ANOVA were used to examine potential group differences in clinical and demographic variables. Subsequently, comparisons between CF, RI, and WM were evaluated by multivariate analysis. A covariance analysis was performed when there were significant differences between groups in variables considered influential in their performance. Independent sample t-tests (Kruskal–Wallis H test) were performed within each clinical group, taking into account sex, comorbidity, OCD subtypes, and medication use. The Pearson correlation was used to analyze the relationship between variables. Cohen’s d (standardized mean differences) was calculated to estimate the magnitude of between-group differences. The meaning of Cohen’s d effect size is the following: d ≥ 0.20 and < 0.50 is small, d ≥ 0.50 and < 0.80 is medium, and d ≥ 0.80 is large. All participants were included in analyses. SPSS Statistic 22.00 (SPSS for windows, Chicago, IL, USA) was used for statistical analysis.

## 3. Results

### 3.1. Group Equivalence

Groups were equivalent in sex (*p* = 0.593), age (*p* = 0.761), educational level (*p* = 0.432), and marital status (*p* = 0.816). The BDI-II scores in the OCD group represent mild/moderate severity. The mean total OBSI-R score among patients with OCD was 190.28. The BAI scores in patients with OCD represent moderate severity. See Table 1.

### 3.2. A Comparison of OCD Group and CG in CF, RI, and WM

Table 2 shows the results of a multivariate analysis (MANOVA) on all variables. The MANOVA test results, based on the Wilks Lambda scale, showed a significant multivariate group effect in the WCST, Corsi-Block Test, Stroop Color–Word Test, and Go/No-Go Task. Significant differences in results of the univariate analysis could be interpreted as signs of impartment of neuropsychological functions among the OCD group, particularly in the WCST the number of categories (*p* = 0.001), perseverative responses (*p* < 0.001), number of errors (*p* < 0.001), perseverative errors (*p* < 0.001), and non-perseverative errors (*p* < 0.001); Corsi SPAN backward (*p* = 0.026); and interference (*p* = 0.017) in the Stroop Color–Word Test; and omissions (*p* = 0.027) and commissions (*p* = 0.040) in the Go/No-Go Task, with the CG obtaining best scores. The effect sizes of measures evaluated with the Stroop Color–Word Test was medium. The WSCT variables were high in magnitude, and the Corsi SPAN backward was medium-low.

### 3.3. CF, RI, and WM Controlling Level of Anxiety, Depression, and Obsessive Beliefs

As anxiety, depression, and OBSI-R variables reached statistically significant differences among groups, covariance analysis was conducted. Depression did not influence results. It was observed that when controlling anxiety and OBSI-R, Corsi backward SPAN no longer showed significant differences (*p* = 0.261). Statistically significant differences in measures were maintained: Stroop interference (*p* = 0.027), number of categories (*p* = 0.015), perseverative responses (*p* = 0.001), number errors (*p* < 0.001), perseverative errors (*p* < 0.001), and non-perseverative errors (*p* < 0.001) of the WCST, with better scores in the CG. 

### 3.4. Intragroup Comparisons Based on Sex, Comorbidity, Medication Use, and OCD Subtypes

There were no sex differences in EF (*p* > 0.05). As for comorbidity, differences were observed in commission errors (*p* = 0.024), Stroop interference (*p* = 0.031), Corsi forward (*p* = 0.022), Corsi backward (*p* = 0.003), total Corsi (*p* = 0.005), Digit SPAN forward (*p* = 0.031), and Digit Scalar Score (*p* = 0.015). Corsi SPAN backward was marginally significant (*p* = 0.051). Participants without a comorbid disorder achieved a higher performance. Medication type influenced the Corsi backward score (*p* < 0.01). Patients who were taking antipsychotics presented the worst scores.

In terms of obsession type, no statistically significant differences were found in any analyzed variables (*p* > 0.05). However, types of compulsions showed differences in the Go/No-Go Task, (*p* = 0.04) in omission errors between wash/clean, repeat and check, with performance being worse in repeaters. In Corsi SPAN forward, the marginally significant differences were presented between repeating and miscellaneous, with repeating patients obtaining better scores (*p* = 0.061).

### 3.5. Correlation between EF, YBOCS Scores, and OCD Duration

The OCD group presented a significant relationship between Y-BOCS scores and Corsi SPAN backward (*r* = −0.359, *p* = 0.05), with a lower performance in this task associated with higher Y-BOCS total scores. There was no significant relationship between OCD duration and EF.

## 4. Discussion

The study’s first aim was to analyze differences in CF, RI, and WM among patients with OCD and a healthy CG. The OCD group presented lower scores in CF, measured with the WCST and the Five Digit Test, coinciding with findings from previous studies [41,42]. In the present study, OCD participants achieved a lower performance both in perseverative errors and number of categories, which are considered the main signs of frontal dysfunction [7]. 

Greater interference was observed in the Stroop and Go/No-Go tasks, indicating lower cognitive and motor RI capacity, consistent with previous findings [13,43]. The failures in error processing and inhibitory control implementation may underlie deficits in stopping unwanted compulsive behaviors in the disorder. 

Similar to other studies [18,44,45], OCD patients performed worse on tasks assessing visuospatial WM as indicated by the Corsi SPAN backward. Perna et al. [45] found that OCD patients had a lower spatial storage capacity, which could contribute to mistrust in memory and a larger dependence on external validations [46]. Martoni et al. [44] concluded that deficits found in visuospatial WM in OCD appeared more severe as workload increased, and that they could be mediated by task strategy (executive dysfunction). ES achieved in CF were high, while medium-low in visuospatial WM and low in cognitive RI [11,12,43]. 

Our second aim was to assess whether differences observed could be due to anxiety, depression, and obsessive beliefs. Results remained the same in CF and RI cognitive, but no differences were found between the OCD and control groups in visuospatial WM. Thus, these results suggest that anxiety and obsessive beliefs may influence verbal RI and WM tasks [29,30]. Conversely, depression did not play a moderating role, which is consistent with Abramovitch and Cooperman [47].

The third aim was to verify EF differences within the OCD group due to sex, comorbidity, medication use, and OCD subtypes. In CF, there were no differences between OCD patients related to these variables, coinciding with previous studies [48]. Comorbidity influenced motor and cognitive RI, with significant differences in commission errors, Stroop interference, and with higher scores in the CG. Comorbidity was also associated with differences in verbal and visuospatial WM, with participants without comorbid disorders achieving higher performance. These results are in line with those suggested by other authors [49]. Nevertheless, medication use influenced visuospatial WM, with better performance in patients who did not take medication. This finding suggests that some psychotropic drugs may have cognitive side effects. However, medication intake did not influence CF and RI in the current study, which contradicts results of previous studies [12,50] and coincides with others [51]. This could be for several reasons, such as that OCD patients without medication could have previously been without medication which may have somehow affected their cognitive functioning permanently; the comparison between medicated vs. non-medicated has not taken into account the type of drug, or the fact that the OCD sample was heterogeneous in the different subtypes of obsession/compulsion [52] (Stein et al., 2008). All these aspects could not be analyzed due to the small sample size.

As for predominant symptoms in OCD patients, no significant differences were observed in EF depending on the type of predominant obsession. However, differences in motor RI (errors of omission) were found when looking at compulsion subtypes among patients with different compulsions, with lower performance in repeaters. In addition, marginally significant differences were observed in visuospatial WM among repeat patients. Those with other compulsions, such as washing or checking, performed better in these tasks. This finding is consistent with Berlin and Lee [53], who observed a relationship between RI and compulsions only, supporting the notion that compulsions could be a behavioral demonstration of underlying RI deficits [54]. Due to the small sample size of the different subtypes, caution must be exercised when drawing conclusions from these results, although a possible differential pattern of EF appears to be evident depending on the predominant OCD compulsion subtype.

The final aim was to examine the relationship between EF, Y-BOCS scores, and OCD duration. Significant relationships were found between the total score in obsessions and visuospatial WM, with lower EF performance associated with higher Y-BOCS scores. Results obtained coincide with those by Abramovitch et al. [25], who found a small degree of negative association between visuospatial memory and symptomatic severity of OCD. We highlight that, in this study, severity at time of test was evaluated, controlling for one of the variables that could influence results. 

As for clinical implications, findings from this study may suggest that incorporating a CF component within treatment could enable more effective interventions. The inclusion of specific modules on flexibility could potentially enhance the effectiveness of exposure with response prevention, improving adherence to treatment, and preventing desertion [55].

This study has some limitations, such as the non-random selection of participants, design type (cross-sectional), and the use of only one evaluation tool for each variable. 

In future studies, it would be interesting to compare our results with pediatric OCD. 

## 5. Conclusions

The control group performed better than the OCD group in tasks involving cognitive flexibility, inhibition, and visuospatial working memory. Deficits in these subdomains may be endophenotypes of OCD.

OCD severity, comorbidity patterns, anxiety, and obsessive beliefs may influence performance.

## Figures and Tables

**Table 1:** Sample measures.

Characteristics	OCD (*n* = 32)	CG (*n* = 30)	T/χ^2^
Age (Mean ± SD)	32.81 ± 8.22	33.53 ± 10.32	ns
Sex *n* (%)			ns
Men	15 (47)	12 (40)	
Women	17 (53)	18 (60)	
Marital status *n* (%)			ns
Single	17 (53.1)	14 (46.7)	
Married	13 (40.6)	15 (50)	
Divorced	2 (6.3)	1 (3.3)	
Educational level *n* (%)			ns
Elementary	5 (15.6)	3 (10)	
Secondary education	7 (21.9)	4 (13.3)	
High school	8 (25)	8 (26.7)	
University student	12 (37.5)	15 (50)	
Type of medication		-	
None Antidepressant	14 (50) 16 (44.6)		
Antipsychotic Antidepressant + antipsychotic	1 (2.7) 1 (2.7)		
BAI (Mean ± SD)	18.91 ± 10.54	7.27 ± 6.04	T (1,62) = 5.377; *p* < 0.001
BDI (Mean ± SD)	19.72 ± 11.98	6.33 ± 4.51	T (1,62) = 5.926; *p* < 0.001
OBSI-R (Mean ± SD)	190.28 ± 47.35	134.10 ± 46.25	T (1,62) = 4.721; *p* < 0.001

*n* = number; SD: standard deviation; ns: not significant. BDI: Beck-II Depression Inventory; BAI: Beck Anxiety Inventory; OBSI-R: Obsessive Beliefs Spanish Inventory-Revised.

**Table 2:** MANOVA and ES in CF, IR, and WM.

	VD	Group	*n*	Mean	SD	F	d Cohen *	
WCST	Number of categories	OCD	32	5.03	1.45	F(1,60) = 13.42, *p* = 0.001	OCD-CG	−0.93
		CG	30	6.00	0.00			
	Perseverative responses	OCD	32	39.09	11.34	F(1,60) = 23.00, *p* < 0.001	OCD-CG	−1.22
		CG	30	50.90	7.53			
	Number errors	OCD	32	38.72	9.02	F(1,60) = 43.78, *p* < 0.001	OCD-CG	−1.68
		CG	30	51.00	4.83			
	Perseverative errors	OCD	32	38.53	10.72	F(1,60) = 27.79, *p* < 0.001	OCD-CG	−1.34
		CG	30	50.50	6.50			
	Non perseverative errors	OCD	32	40.31	7.15	F(1,60) = 45.55, *p* < 0.001	OCD-CG	−1.72
		CG	30	50.73	4.65			
Corsi block	Forward score	OCD	32	10.81	2.40	F(1,60) = 0.03, *p =* 0.858	OCD-CG	−0.04
		CG	30	10.93	2.89			
	Backward score	OCD	32	8.91	3.00	F(1,60) = 1.67, *p* = 0.202	OCD-CG	−0.32
		CG	30	9.83	2.63			
	Forward SPAN	OCD	32	11.12	2.70	F(1,60) = 0.59, *p* = 0.447	OCD-CG	0.19
		CG	30	10.60	2.70			
	Backward SPAN	OCD	32	8.91	3.00	F(1,60) = 5.19, *p* = 0.026	OCD-CG	−0.32
		CG	30	9.83	2.63			
	Total score	OCD	32	16.94	3.01	F(1,60) = 1.84, *p* = 0.180	OCD-CG	−0.34
		CG	30	18.07	3.53			
Digits test	SPAN forward	OCD	32	6.47	1.27	F(1,60) = 0.24, *p* = 0.626	OCD-CG	0.12
		CG	30	6.30	1.44			
	SPAN backward	OCD	32	4.91	1.20	F(1,60) = 0.40, *p* = 0.53	OCD-CG	−0.15
		CG	30	5.13	1.61			
	SPAN increasing	OCD	32	5.78	1.24	F(1,60) = 0.12, *p* = 0.731	OCD-CG	0.08
		CG	30	5.67	1.37			
	Scalar score	OCD	32	10.00	3.25	F(1,60) = 0.18, *p* = 0.672	OCD-CG	−0.11
		CG	30	10.37	3.54			
Stroop C/W	Stroop Interference	OCD	32	49.19	7.53	F(1,60) = 6.04, *p* = 0.017	OCD-CG	−0.62
		CG	30	53.80	7.23			
Go/No-Go	Omissions errors	OCD	32	1.22	2.29	F(1,60) = 5.79, *p* = 0.027	OCD-CG	−0.53
		CG	30	0.23	1.22			
	Commission errors	OCD	32	2.47	2.01	F(1,60) = 4.39, *p* = 0.040	OCD-CG	−0.46
		CG	30	1.67	1.35			

OCD: obsessive–compulsive disorder; CG: control group. ES: effect size. * Negative *Ds* indicated that the group compared in first place reached the worst score achieved by the group appearing in second place.

## Data Availability

The data that support the findings of this study are available on request from the corresponding author.
